# Long-Term Sick Leave for Psychiatric Disorders Among Healthcare Workers

**DOI:** 10.1192/j.eurpsy.2025.1477

**Published:** 2025-08-26

**Authors:** M. Makhloufi, M. Bouhoula, N. Gannoun, A. Ghenim, B. Narjes, A. Chouchene, A. Aloui, I. Kacem, M. Maoua, H. Kalboussi, O. El Maalel, A. Brahem, S. Chatti

**Affiliations:** 1 Occupational Medicine Department, Farhat Hached University Hospital; 2 Department of Occupational Medicine and Occupational Pathology; 3 Department of Occupational Medicine and Occupational Pathology, Faculty of Medicine of Sousse; 4 Occupational Medicine Department, Sahloul University Hospital, sousse, Tunisia

## Abstract

**Introduction:**

Absenteeism is a significant issue affecting various professional sectors, and its increase within healthcare institutions is a major concern. This phenomenon is complex and multidimensional, involving numerous factors.

**Objectives:**

To determine the rate of absenteeism among healthcare personnel, describe the socioprofessional characteristics of employees on long-term sick leave due to psychiatric disorders, and identify the main factors associated with this absenteeism.

**Methods:**

This is a retrospective descriptive study conducted on all healthcare personnel in a Tunisian governorate who took long-term sick leave for psychiatric reasons between 2015 and 2020. Data were collected from medical and administrative records through the Regional Long-Term Sick Leave Commission and via a questionnaire conducted by phone

**Results:**

Among the 5067 employees in healthcare facilities in the governorate, 388 (7.65%) had taken at least one period of sick leave due to psychiatric issues. The average age of absent personnel was 45 ± 9 years, with a range of 30 to 65 years. A significant majority of the absentees were female, accounting for 85.8%. Additionally, 78.8% of the absent employees were married. University hospitals (UHs) accounted for 75.6% of absenteeism cases, with nurses being the most affected professional group (40.5%). The duration of sick leave ranged from 1 to 60 months, with an average of 8.5 months. Depression was the most frequent psychiatric cause, accounting for 82.34% of cases. The analysis showed that depression was significantly associated with several factors, notably age (p=0.021), job position (p=0.049), and employment in UHs (p=0.027).

**Conclusions:**

The results indicate high levels of absenteeism, emphasizing the urgent need for a comprehensive intervention plan. This plan should prioritize preventive measures, targeting both individuals and workplace organization.

**Disclosure of Interest:**

None Declared

